# Supplementary material for: Detection of Tuberculosis by The Analysis of Exhaled Breath Particles with High-resolution Mass Spectrometry
Source: Sci Rep. 2020 May 6;10:7647. doi: 10.1038/s41598-020-64637-6 (PMC7203136; doi:10.1038/s41598-020-64637-6)
Supplement: Supplementary file 2 — Supplementary information 2. [file 41598_2020_64637_MOESM2_ESM.pdf]

Supporting Information

Detection of Tuberculosis by The Analysis of Exhaled Breath Particles with High-resolution Mass Spectrometry

Dapeng Chen[1]*, Wayne A. Bryden[1], Robin Wood[2]

[1]Zeteo Tech Inc, Sykesville, Maryland, United States of America; [2]Desmond Tutu HIV Centre, Institute of Infectious Diseases and Molecular Medicine, University of Cape Town, Cape Town, South Africa

*To whom correspondence should be addressed: dapeng.chen@zeteotech.com

Supplementary Figure:

Figure S1. High-resolution mass spectrometry analysis of lipid standards.

Supplementary Tables:

Table S1. Active TB patient and non-TB subject information.

Table S2. Raw data files of SAM analysis of features extracted from positive ion and negative ion mode.

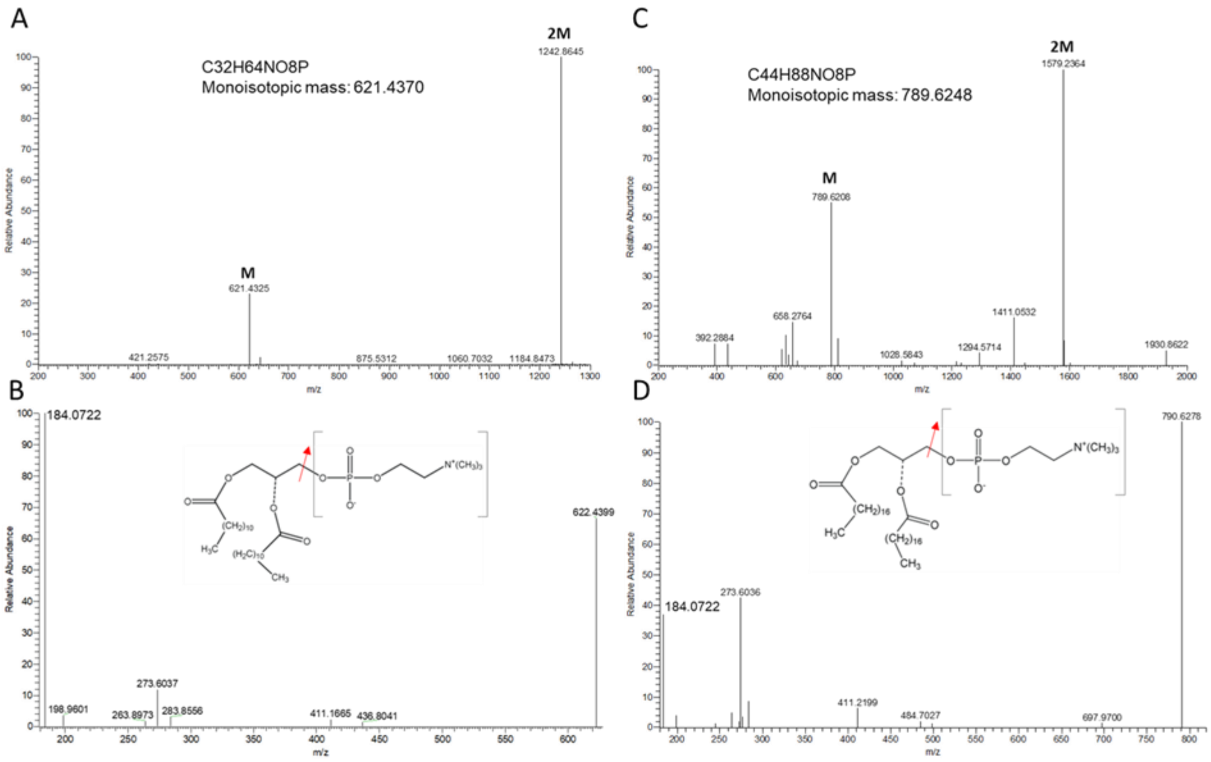

Fig. S1. High-resolution mass spectrometry analysis of lipid standards. Mass spectra of precursor (A, C) and fragment ion patterns (B, D) of two lipid standards, 1,2-Distearoyl-sn-glycero-3-phosphorylcholine (C44H88NO8P) and 1,2-Dilauroyl-sn-glycero-3-phosphorylcholine (C32H64NO8P). Ion fragmentation was supported by higher-energy collision dissociation (HCD) with 35% of energy. The precursor isolation window was defined as 3 m/z. Both phospholipids demonstrate fragmentations between phosphoryl group and ester (phospho(di-)ester linkage, indicated by red arrows) and produce an identical diagnostic fragment ion of phosphorylcholine (m/z 184.07, B, D, Ref 1).

Ref 1. Pulfer M1, Murphy RC. Electrospray mass spectrometry of phospholipids. Mass Spectrom Rev. 2003 Sep-Oct;22(5):332-64
